# Postoperative Delirium in Patients with Chronic Obstructive Pulmonary Disease after Coronary Artery Bypass Grafting

**DOI:** 10.3390/medicina56070342

**Published:** 2020-07-09

**Authors:** Aleksandra Szylińska, Iwona Rotter, Mariusz Listewnik, Kacper Lechowicz, Mirosław Brykczyński, Sylwia Dzidek, Maciej Żukowski, Katarzyna Kotfis

**Affiliations:** 1 Department of Medical Rehabilitation and Clinical Physiotherapy, Pomeranian Medical University in Szczecin, Żołnierska 48, 71-210 Szczecin, Poland; aleksandra.szylinska@gmail.com (A.S.); iwrot@wp.pl (I.R.); 2 Department of Cardiac Surgery, Pomeranian Medical University in Szczecin, al. Powstańców Wielkopolskich 72, 70-111 Szczecin, Poland; mariusz.listewnik@pum.edu.pl (M.L.); miroslaw.brykczynski@pum.edu.pl (M.B.); 3 Department of Anesthesiology, Intensive Therapy and Acute Intoxications, Pomeranian Medical University in Szczecin, Al. Powstańców Wielkopolskich 72, 70-111 Szczecin, Poland; kacper.lechowicz@gmail.com (K.L.); zukowski@pum.edu.pl (M.Ż.); 4 Student Science Club at the Department of Anesthesiology, Intensive Therapy and Acute Intoxications, Pomeranian Medical University, 70-111 Szczecin, Poland; sylwia.dzidek@wp.pl

**Keywords:** COPD, cardiac surgery, CABG, delirium, POD, CAM-ICU

## Abstract

Background and Objectives: The incidence of postoperative delirium (POD) in patients with chronic obstructive pulmonary disease (COPD) is unclear. It seems that postoperative respiratory problems that may occur in COPD patients, including prolonged mechanical ventilation or respiratory-tract infections, may contribute to the development of delirium. The aim of the study was to identify a relationship between COPD and the occurrence of delirium after cardiac surgery and the impact of these combined disorders on postoperative mortality. Materials and Methods: We performed an analysis of data collected from 4151 patients undergoing isolated coronary artery bypass grafting (CABG) in a tertiary cardiac-surgery center between 2012 and 2018. We included patients with a clinical diagnosis of COPD according to the Global Initiative for Chronic Obstructive Lung Disease (GOLD) criteria. The primary endpoint was postoperative delirium; Confusion Assessment Method in the Intensive Care Unit (CAM-ICU) was used for delirium assessment. Results: Final analysis included 283 patients with COPD, out of which 65 (22.97%) were diagnosed with POD. Delirious COPD patients had longer intubation time (*p* = 0.007), more often required reintubation (*p* = 0.019), had significantly higher levels of C-reactive protein (CRP) three days after surgery (*p* = 0.009) and were more often diagnosed with pneumonia (*p* < 0.001). The CRP rise on day three correlated positively with the occurrence of postoperative pneumonia (r = 0.335, *p* = 0.005). The probability of survival after CABG was significantly lower in COPD patients with delirium (*p* < 0.001). Conclusions: The results of this study confirmed the relationship between chronic obstructive pulmonary disease and the incidence of delirium after cardiac surgery. The probability of survival in COPD patients undergoing CABG who developed postoperative delirium was significantly decreased.

## 1. Introduction

Coronary artery bypass grafting (CABG) is one of the most frequently performed heart operations worldwide. In 2016, 54% of all heart surgeries recorded in The Society of Thoracic Surgeons database were isolated CABG procedures, and CABG combined with valve replacements comprised an additional 8% [1]. In addition to coronary artery disease, patients undergoing these procedures also have a number of age-related associated illnesses, such as hypertension, diabetes, atrial fibrillation and obstructive pulmonary disease [2,3]. The postoperative outcome after CABG relates to this multimorbidity and may be associated with many complications, including respiratory failure, stroke, postoperative delirium (POD) or acute kidney injury.

Chronic obstructive pulmonary disease (COPD) is estimated to be the third-leading cause of death in the world [4]. Depending on the population, its incidence ranges between 8% and 20% [5]. COPD is characterized by chronic inflammation of the respiratory tract with an irreversible restriction of the airway lumen [6]. The main cause of COPD is smoking [7]. It has been proven that COPD is associated with the presence of cardiovascular disease [8]. COPD is triggered by persistent inflammation causing remodeling of the airways. Excessive activation of inflammatory cells affects the whole body, not only the lung tissue, and also contributes to the formation of atherosclerosis, which leads to irreversible changes in the arterial vessels, including those of the heart and the brain [8].

One of the common neurological complications of cardiac surgery is postoperative delirium. According to the American Psychiatric Association (APA), delirium is defined as a disturbance of consciousness, with reduced clarity of awareness of the environment and reduced ability to focus, sustain or shift attention [9]. This acute pathology of the central nervous system is often reversible and may be associated with a worse postoperative condition of the patient after cardiac surgery [10,11]. Postoperative delirium is associated with increased mortality but also extends the duration of hospitalization and intensive-care-unit (ICU) stay [12]. It also increases the risk of developing postoperative cognitive disorders [13,14,15].

Although ICU delirium is a serious diagnostic and therapeutic problem with an estimated frequency between 60% and 80% in mechanically ventilated patients [15], it is often underdiagnosed [16]. The attention of many scientists is focused on searching for factors related to the appearance of POD, which could potentially improve the prognosis of patients after major cardiac surgery. In the search for answers, it is worth focusing on the pathophysiology of the delirium formation process. It is suggested that the neuroinflammation affecting the nervous system and glial-cell activation plays a major role. There are many factors associated with it. CABG operations usually take place using cardiopulmonary bypass (CPB), which activates inflammatory mediators, affecting microglial cells in the central nervous system and triggering delirium [17,18]. Among the previously proven risk factors for delirium are old age, type of surgery, history of psychiatric disorders, renal failure, postoperative atrial fibrillation, time of mechanical ventilation, blood transfusion, cardiovascular disease, metabolic disorders and blood oxygenation during and after surgery [19]. Another problem specifically relevant in patients with COPD may be related to inadequate tissue oxygenation but also to the need for prolonged intubation, mechanical ventilation and, thus, sedation or postoperative respiratory infections [4,20].

To date, no previous analysis has been conducted to specifically determine the incidence of postoperative delirium in COPD patients. It seems that postoperative respiratory disorders that occur in COPD patients may contribute to the worsening of the mental condition. Moreover, respiratory failure in COPD patients has been shown to intensify myocardial incidents in the form of infarction and contribute to increased mortality [21].

The aim of the study was to identify the relationship between preoperative chronic obstructive pulmonary disease and the occurrence of postoperative delirium in patients undergoing cardiac surgery and the impact of these disorders combined on postoperative mortality.

## 2. Materials and Methods

### 2.1. Study Population

A retrospective cohort analysis of prospectively collected data was carried out and included 4151 patients that were qualified for an isolated coronary artery bypass grafting procedure using CPB at the Department of Cardiac Surgery at Pomeranian Medical University in Szczecin, Poland (a tertiary cardiac surgery center) between 1 January 2012 and 31 December 2018.

**Figure 1:**
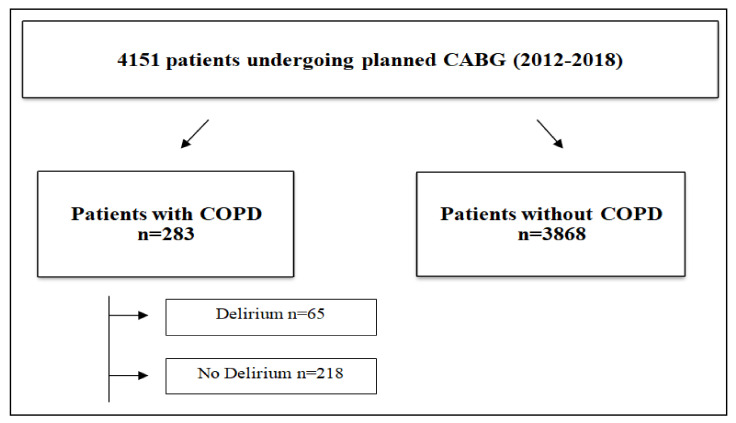
Study flowchart. Legend: CABG—Coronary artery bypass grafting; COPD—chronic obstructive pulmonary disease.

The whole collected cohort consisted of 6998 patients undergoing cardiac surgery at our department during that time. Subsequently, we included only patients undergoing isolated coronary artery bypass grafting (n = 4151). The patients were divided into two groups depending on the presence or absence of COPD diagnosis. The group with COPD was additionally divided into two subgroups: patients with (n = 65) or without (n = 218) an incidence of postoperative delirium (Figure 1).

### 2.2. Data Collection

We reviewed the medical documentation for demographic data (age, sex, body mass index (BMI) and smoking) and preoperative data (Canadian Cardiovascular Society (CCS) score, New York Heart Association (NYHA) score, ejection fraction and laboratory testing). We used the EuroScore Logistics 2 scale to calculate the perioperative risk for each patient [22]. We also collected patients’ history before admission, which included standard data: stroke, transient ischemic attack (TIA), epilepsy, chronic renal failure, internal carotid artery, glucose intolerance, diabetes, arterial hypertension, thyroid disease, acute myocardial infarction, atrial fibrillation, hyperlipidemia and peripheral vascular disease. Moreover, we collected information regarding the intraoperative and postoperative period, which included standard laboratory data, cardiopulmonary bypass time, aortic cross-clamping time, reperfusion time, the use of hemofiltration, number of coronary grafts, endotracheal intubation time, reintubation incidence and time and time of death after surgery. Patients with a clinical diagnosis of COPD according to the Global Initiative for Chronic Obstructive Lung Disease (GOLD) criteria stages I to IV were included in the analysis [23]. The primary endpoint was the presence of postoperative delirium. All patients were screened for the presence of delirium twice a day using the Confusion Assessment Method in the Intensive Care Unit (CAM-ICU) tool as per study protocol, but the final diagnosis was made according to the DSM V criteria [9].

### 2.3. Ethical Statement

Due to the retrospective nature of the study, it received a waiver from the Bioethical Committee of the Pomeranian Medical University in Szczecin, Poland (decision no. KB-0012/277/10/18). The study was performed in accordance with the Declaration of Helsinki. Before the intervention, all participants signed written informed consent as part of routine preoperative document workup and were notified that they could withdraw their consent from the trial at any point without any consequences. The documents included consent for data collection and medical-record review. All data were dehumanified to ensured confidentiality and anonymity of the patients.

### 2.4. Statistical Analysis

Data analysis was performed using Statistica 13 licensed software (StatSoft, Inc., Tulsa, OK, USA). To characterize the study population, we used descriptive statistics. All the continuous-variable data are presented as the mean and standard deviation (SD); categorical variables are presented as proportions. The chi-square test or chi-square test with Yates’s correction was used to compare qualitative data between the two groups of patients. The normality of the data distribution was checked using the Shapiro–Wilk test. Differences in continuous variables between groups were tested using Mann–Whitney U tests. We performed a univariate logistic regression analysis for patients with COPD. Kaplan–Meier analysis calculated the probability of survival. Statistical significance was accepted at *p* < 0.05 for all comparisons. 

## 3. Results

### 3.1. Relationship between COPD and Delirium

Postoperative delirium was analyzed depending on the incidence of COPD among all 4151 patients who underwent coronary artery bypass surgery. Delirium was observed in 65 patients (22.97%) with COPD (*p* = 0.015), the data is visible in Table 1.

Logistic regression was performed to confirm the analysis, and it showed a significant increase in the incidence of delirium in COPD patients (OR = 1.428, Cl = 1.07–1.91, *p* = 0.016).

### 3.2. Clinical Characteristics of Patients with COPD

The final analysis included 283 patients with diagnosed COPD. The analysis of demographic data found no significant differences between the groups. The only significant difference was a higher EuroScore Logistics 2 score in delirious patients (*p* = 0.041). Demographic data and clinical characteristics are shown in Table 2.

The analysis of intraoperative and postoperative data showed differences between patients with and without delirium (Table 3). Delirious COPD patients had longer intubation time (*p* = 0.007), more often required reintubation (*p* = 0.019), had significantly higher levels of C-reactive protein (CRP) three days after surgery (*p* = 0.009) and were more often diagnosed with pneumonia (*p* < 0.001). The CRP rise on day three correlated positively with the occurrence of postoperative pneumonia, r = 0.335, *p* = 0.005. In this group, more hospital deaths occurred (*p* = 0.009).

### 3.3. Survival Analysis

**Figure 2:**
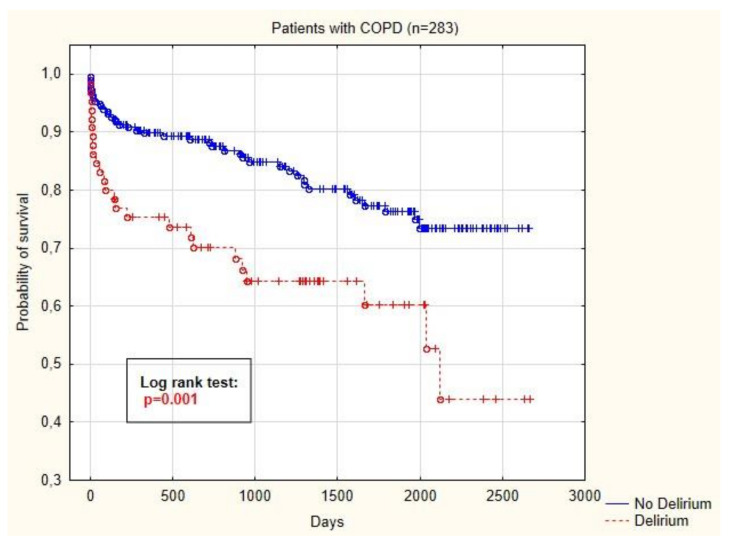
Kaplan–Meier curve showing probability of survival of COPD patients depending on the presence of postoperative delirium.

The Kaplan–Meier curve is visible in Figure 2 and shows that the probability of survival after CABG was significantly lower in COPD patients with delirium (*p* < 0.001).

## 4. Discussion

In the current study, we performed an analysis of a large homogenous cohort of patients undergoing an isolated coronary artery bypass grafting procedure and found that patients who were diagnosed with COPD before surgery were more likely to have delirium after surgery. The results of this study confirmed the relationship between preoperative chronic obstructive pulmonary disease and the incidence of delirium after surgery. The probability of survival in patients with COPD and delirium was significantly decreased.

Studies have shown that COPD alone is an independent predictor of hospital mortality in patients after CABG [24,25,26,27,28,29]. Huang et al. showed that this group of patients had an increased perioperative risk and mortality, regardless of the performed coronary intervention of percutaneous coronary intervention (PCI) or CABG [24]. Many studies describe an increased surgical risk in patients with obstructive pulmonary lesions, particularly among patients undergoing cardiac surgery. Obstructive pulmonary changes confirmed by spirometry are described as a factor predicting the patient’s prolonged stay in the hospital and increased mortality [30,31,32]. The authors suggest that the results of spirometry analysis should be included in the stratification of perioperative risk for patients undergoing CABG. 

The occurrence of postoperative delirium is a significant problem in postoperative care. It is estimated that after cardiac surgery, the incidence is between 16% and 73%. However, it is often not recognized by medical personnel [16,33]. The occurrence of delirium is influenced by many factors that can be divided into preoperative, perioperative and postoperative. Preoperative risk factors of POD include patient age and comorbidities such as diabetes, atherosclerosis, myocardial infarction and water and electrolyte disturbances [15,33]. In our research, we collected data regarding a homogeneous group of patients in terms of demographic and preoperative data and regarding comorbidities; the only difference was the EuroScore Logistics scale, which includes chronic lung disease in the risk assessment. The obtained results showed a statistically significant relationship between the occurrence of POD and COPD. Despite the lack of large analyses regarding this issue, available literature may confirm this relationship. In a retrospective study conducted by Cui et al., COPD was an independent risk factor for delirium among patients undergoing spine surgery [34]. Other studies assessing the condition of ICU patients have indicated that COPD is an independent risk factor for delirium (OR = 3.5, *p* = 0.005) [35]. Austin et al. showed a relationship between the onset of stroke and systemic inflammation and oxidative stress in COPD [36]. Lahousse et al. explored the topic and showed a relationship between the occurrence of COPD and cerebrovascular disease, including cognitive impairment, but without specifying delirium [37]. 

In the pathogenesis of delirium and other neurological consequences of cardiac and vascular surgery, many factors can be distinguished, including systemic inflammation and blood-oxygenation disorders [18,38,39]. Both of these factors occur in COPD patients, which may contribute to an increased incidence of delirium in this population. Gosselt et al. performed a large mega-analysis assessing risk factors for delirium in the postoperative period after heart surgery, listing factors such as age, preadmission mental state, cerebrovascular disease and postoperative blood-oxygenation disorders [40]; however, no significance was shown regarding COPD patients.

It is worth mentioning that the use of cardiopulmonary bypass (CPB) may be associated with an increased risk of postoperative delirium in cardiac surgery. CPB activates inflammatory mediators that affect microglial cells in the central nervous system, thus triggering delirium [17,18]. It has been shown that in operations using CPB, there is an increased level of biomarkers of neuronal damage, such as tau protein and neurofilament light protein. This has correlated positively with the occurrence of postoperative delirium [41,42].

The occurrence of a delirium episode has its distant complications. One such complication is increased mortality. Reduced long-term survival can be seen in COPD patients who have delirium. This observation is consistent with the data available in the literature. Gottesman et al. conducted an analysis of their patients undergoing CABG surgery and observed that a delirium episode may involve an increased hazard of death (HR = 1.65) up to 10 years after surgery [43]. Therefore, it is critical to follow international guidelines to limit the occurrence of delirium, including the use of the ABCDEF bundle (A—Assess, Prevent and Manage Pain; B—Both Spontaneous Awakening Trials (SAT) and Spontaneous Breathing Trials (SBT); C—Choice of Analgesia and Sedation; D—Delirium: Assess, Prevent and Manage; E—Early Mobility and Exercise; F—Family Engagement and Empowerment) [41]. Nonpharmacological interventions included in the ABCDEF bundle are the mainstay of effective delirium prophylaxis and treatment. Those activities focus on adequate pain monitoring and treatment, minimization of sedation and mechanical ventilation, monitoring and treatment of delirium, early introduction of physiotherapy and involvement of family in patient care [44,45,46]. Further studies are necessary to evaluate the effects of pre-rehabilitation and early postoperative physiotherapy on the incidence of postoperative delirium in COPD patients.

## 5. Limitations

One of the main limitations of our study is its single-center character, so the results relate only to patients from a given region. Another limitation of the study is the lack of spirometry-test results. The addition of spirometry would allow for stratifying delirium according to COPD severity.

## 6. Conclusions

The results of this study confirmed the relationship between chronic obstructive pulmonary disease and the incidence of delirium after coronary artery bypass surgery. The probability of survival in COPD patients with delirium was significantly decreased.

## Figures and Tables

**Table 1:** Assessment of the occurrence of delirium in patients diagnosed with chronic obstructive pulmonary disease (COPD) and without COPD.

	No COPD (n = 3868)		COPD (n = 283)		*p*-Value
	n	%	n	%	
**No Delirium**	3199	82.73%	218	77.03%	0.015
**Delirium**	668	17.27%	65	22.97%	

**Table 2:** Demographic and preoperative data characteristics for COPD patients with and without delirium.

Variables		COPD Patients (n = 283)		
		No Delirium (n = 218)	Delirium (n = 65)	*p*
**Demographic data**				
**Age (years), Me (Q1–Q3)**		66.0 (62.0–72.0)	68.0 (63.0–73.0)	0.335
**Female, n (%)**		54 (24.77)	23 (35.38)	0.091
**BMI (kg/m^2^), Me (Q1–Q3)**		29.3 (26.2–32.4)	29.4 (25.3–31.9)	0.957
**Smoking, n (%)**	never smoked	44 (20.28)	8 (12.31)	0.149
	ex–smoker	141 (64.98)	42 (64.62)	
	current smoker	32 (14.75)	15 (23.08)	
**Smoking (years), Me (Q1–Q3)**		40.0(30.0–45.0)	40.0 (30.0–50.0)	0.368
**Preoperative data**				
**EuroScore Logistic II (%), Me (Q1–Q3)**		2.2 (1.3–3.5)	2.7 (1.6–3.9)	0.041
**scale CCS, Me (Q1–Q3)**		3.0 (3.0–4.0)	3.0 (2.0–3.0)	0.081
**scale NYHA, Me (Q1–Q3)**		2.0 (2.0–3.0)	2.0 (2.0–3.0)	0.522
**Ejection Fraction (%), Me (Q1–Q3)**		50.0 (40.0–55.0)	45.0 (35.0–50.0)	0.079
**CKMB (µg/L), Me (Q1–Q3)**		21.0 (18.0–26.5)	22.00 (18.0–27.0)	0.921
**CRP (mg/L), Me (Q1–Q3)**		2.8 (1.0–5.7)	2.2 (1.2–4.5)	0.649
**Glycated hemoglobin (%), Me (Q1–Q3)**		5.8 (5.5–6.3)	5.9 (5.6–6.6)	0.199
**Creatinine (mg/dL), Me (Q1–Q3)**		0.9 (0.8–1.1)	0.9 (0.8–1.1)	0.743
**GFR (mL/min/1.73 m^2^), Me (Q1–Q3)**		80.0 (63.0–92.0)	76.0 (62.0–91.0)	0.569
**Co-morbidities**				
**Stroke, n (%)**		9 (4.13)	1 (1.54)	0.321
**TIA/RIND, n (%)**		4 (1.83)	2 (3.08)	0.542
**CCS IV, n (%)**		59 (27.06)	12 (18.46)	0.160
**NYHA III and IV, n (%)**		30 (13.76)	9 (13.85)	0.851
**ICA stenosis, n (%)**		11 (5.05)	5 (7.69)	0.614
**Chronic Renal Failure, n (%)**		11 (7.59)	8 (17.02)	0.109
**Glucose intolerance, n (%)**		6 (2.75)	2 (3.08)	0.774
**Diabetes, n (%)**		74 (33.94)	30 (46.15)	0.077
**Arterial hypertension, n (%)**		169 (77.52)	53 (81.54)	0.489
**Thyroid disease, n (%)**		17 (7.80)	8 (12.31)	0.381
**AF paroxysmal, n (%)**		27 (12.39)	5 (7.69)	0.409
**AF persistent or permanent, n (%)**		9 (4.13)	1 (1.54)	0.542
**Hyperlipidemia, n (%)**		73 (33.49)	26 (40.00)	0.334
**Peripheral vascular disease, n (%)**		69 (31.65)	22 (33.85)	0.739

Abbreviations: COPD—chronic obstructive pulmonary disease; BMI—body mass index (kg/m^2^); NYHA—New York Heart Association; CCS—Canadian Cardiovascular Society; CKMB—creatine kinase myocardial band; CRP—C-reactive protein; TIA—transient ischemic attack; EPI—epilepsy; ICA—internal carotid artery; AF—atrial fibrillation; HbA1c—glycated hemoglobin; GFR—glomerular-filtration rate; n—number of patients; Me—median; Q1—first quartile; Q3—third quartile; p—statistical significance.

**Table 3:** Baseline intraoperative and postoperative data for COPD patients with and without delirium.

Variables		COPD Patients (n = 283)		
		No Delirium (n = 218)	Delirium (n = 65)	*p*
**Intraoperative data**				
CPB time (min), Me (Q1–Q3)		50.0 (42.0–60.0)	52.0 (45.0–66.0)	0.198
Aortic cross-clamping time, Me (Q1–Q3)		30.0 (24.0–36.0)	30.0 (26.0–37.5)	0.394
Number of grafts, Me (Q1–Q3)		3.0 (2.0–4.0)	3.00 (2.0–3.0)	0.553
Hemofiltration on CPB, n (%)		37 (16.97)	18 (27.69)	0.055
Hemofiltration (mL), Me (Q1–Q3)		1500.0 (1300.0–1800.0)	1850.0 (1500.0–2000.0)	0.123
Intubation time (min), Me (Q1–Q3)		645.0 (490.0–850.0)	775.0 (585.0–1050.0)	0.007
Re-intubation, n (%)		10 (4.59)	9 (13.85)	0.019
Re-intubation time (min), Me (Q1–Q3)		49.0 (5.0–60.0)	88.00 (36.0–100.0)	0.307
**Postoperative data**				
CKMB (µg/L), Me (Q1–Q3)		42.0 (34.0–57.0)	48.0 (36.0–100.0)	0.053
Max CKMB > 120		4 (1.83)	3 (4.62)	0.205
Acute myocardial infarction, n (%)		4 (1.83)	2 (3.08)	0.542
Low Output syndrome		4 (1.83)	3 (4.62)	0.205
Intensive Care Unit length of stay (days), Me (Q1–Q3)		2.0 (2.0–2.0)	2.0 (2.0–2.0)	0.946
Hemofiltration in the ICU, n (%)		19 (8.72)	9 (13.85)	0.221
RBC (units), Me (Q1–Q3)		2.0 (2.0–2.0)	2.0 (2.0–4.0)	0.094
RBC (n, %)		70 (32.11)	33 (50.77)	0.006
Plasma (units), Me (Q1–Q3)		3.0 (3.0–3.0)	3.0 (3.0–3.0)	0.845
Plasma (n, %)		35 (16.06)	12 (18.46)	0.647
PLR (mL), Me (Q1–Q3)		300.0 (250.0–500.0)	300.0 (250.0–600.0)	0.869
PLR (n, %)		42 (19.27)	16 (24.62)	0.446
Drainage (mL), Me (Q1–Q3)		400.0 (262.5–540.0)	420.0 (285.0–625.0)	0.426
CRP after surgery (mg/L), Me (Q1–Q3)	Day 1	74.2 (44.1–94.6)	67.0 (51.1–72.0)	0.497
	Day 2	242.0 (166.0–305.0)	254.0 (219.0–288.0)	0.538
	Day 3	141.5 (94.5–235.5)	226.5 (212.5–278.0)	0.009
	Day 4	111.0 (66.0–158.0)	156.0 (88.0–180.0)	0.102
	Day 5	106.0 (85.5–122.0)	120.0 (82.5–176.5)	0.431
Highest Creatinine (mg/dL), Me (Q1–Q3)		1.1 (0.9–1.4)	1.26 (0.9–1.7)	0.182
Lowest GFR (mL/min/1.73m^2^), Me (Q1–Q3)		66.5 (43.0–86.0)	51.0 (34.0–78.0)	0.144
Pneumonia, n (%)		131 (3.83)	86 (11.73)	<0.001
Hospital mortality, n (%)		12 (5.50)	10 (15.38)	0.009
Length of hospital stay (LOS), Me (Q1–Q3)		8.0 (7.0–9.0)	7.0 (6.0–9.0)	0.219

Abbreviations: CPB—cardiopulmonary bypass; CKMB—creatine kinase myocardial band; RBC—Red-blood-cell concentrate; PLR—platelets, leukocytes reduced; CRP—C-reactive protein; TIA—transient ischemic attack; EPI—epilepsy; ICA—internal carotid artery; AF—atrial fibrillation; HbA1c—glycated hemoglobin; GFR—glomerular-filtration rate; LOS—length of hospital stay; n—number of patients; Me—median; Q1—first quartile; Q3—third quartile; p—statistical significance.

## References

[1] D’Agostino R.S., Jacobs J.P., Badhwar V., Fernandez F.G., Paone G., Wormuth D.W., Shahian D.M. (2018). The Society of Thoracic Surgeons Adult Cardiac Surgery Database: 2018 Update on Outcomes and Quality. Ann. Thorac. Surg..

[2] Licker M., Schweizer A., Ellenberger C., Tschopp J.M., Diaper J., Clergue F. (2007). Perioperative Medical Management of Patients with COPD. Int. J. COPD.

[3] Wada H., Miyauchi K., Daida H. (2019). Gender Differences in the Clinical Features and Outcomes of Patients with Coronary Artery Disease. Expert Rev. Cardiovasc. Ther..

[4] Vestbo J., Hurd S.S., Agustí A.G., Jones P.W., Vogelmeier C., Anzueto A., Barnes P.J., Fabbri L.M., Martinez F.J., Nishimura M. (2013). Global Strategy for the Diagnosis, Management, and Prevention of Chronic Obstructive Pulmonary Disease GOLD Executive Summary. Am. J. Respir. Crit. Care Med..

[5] Menezes A.M.B., Perez-Padilla R., Jardim J.R.B., Muino A., Lopez M.V., Valdivia G., Montes de Oca M., Talamo C., Hallal P.C., Victora C.G. (2005). Chronic Obstructive Pulmonary Disease in Five Latin American Cities (the PLATINO Study): A Prevalence Study. Lancet.

[6] Pauwels R.A., Buist A.S., Calverley P.M., Jenkins C.R., Hurd S.S. (2001). Global Strategy for the Diagnosis, Management, and Prevention of Chronic Obstructive Pulmonary Disease. NHLBI/WHO Global Initiative for Chronic Obstructive Lung Disease (GOLD) Workshop Summary. Am. J. Respir. Crit. Care Med..

[7] Barnes P.J., Shapiro S.D., Pauwels R.A. (2003). Chronic Obstructive Pulmonary Disease: Molecular and Cellular Mechanisms. Eur. Respir. J..

[8] André S., Conde B., Fragoso E., Boléo-Tomé J.P., Areias V., Cardoso J. (2019). COPD and Cardiovascular Disease. Pulmonology.

[9] American Psychiatric Association (2013). Neurocognitive Disorders. Diagnostic and Statistical Manual of Mental Disorders.

[10] Hollinger A., Siegemund M., Goettel N., Steiner L.A. (2015). Postoperative Delirium in Cardiac Surgery: An Unavoidable Menace?. J. Cardiothorac. Vasc. Anesth..

[11] Kotfis K., Szylińska A., Listewnik M., Strzelbicka M., Brykczyński M., Rotter I., Żukowski M. (2018). Early Delirium after Cardiac Surgery: An Analysis of Incidence and Risk Factors in Elderly (≥65 Years) and Very Elderly (≥80 Years) Patients. Clin. Interv. Aging.

[12] Pun B.T., Balas M.C., Barnes-Daly M.A., Thompson J.L., Aldrich J.M., Barr J., Byrum D., Carson S.S., Devlin J.W., Engel H.J. (2019). Caring for Critically Ill Patients with the ABCDEF Bundle: Results of the ICU Liberation Collaborative in Over 15,000 Adults. Crit. Care Med..

[13] Kotfis K., Marra A., Ely E.W. (2018). ICU Delirium—A Diagnostic and Therapeutic Challenge in the Intensive Care Unit. Anaesthesiol. Intensive Ther..

[14] McPherson J.A., Wagner C.E., Boehm L.M., Hall J.D., Johnson D.C., Miller L.R., Burns K.M., Thompson J.L., Shintani A.K., Ely E.W. (2013). Delirium in the Cardiovascular ICU: Exploring Modifiable Risk Factors. Crit. Care Med..

[15] Sockalingam S., Parekh N., Bogoch I.I., Sun J., Mahtani R., Beach C., Bollegalla N., Turzanski S., Seto E., Kim J. (2005). Delirium in the Postoperative Cardiac Patient: A Review. J. Card. Surg..

[16] Marra A., Kotfis K., Hosie A., MacLullich A.M.J., Pandharipande P.P., Ely E.W., Pun B.T. (2019). Delirium Monitoring: Yes or No? That Is the Question. Am. J. Crit. Care Off. Publ. Am. Assoc. Crit. Nurses.

[17] van Harten A.E., Scheeren T.W.L., Absalom A.R. (2012). A Review of Postoperative Cognitive Dysfunction and Neuroinflammation Associated with Cardiac Surgery and Anaesthesia. Anaesthesia.

[18] Rudolph J.L., Ramlawi B., Kuchel G.A., McElhaney J.E., Xie D., Sellke F.W., Khabbaz K., Levkoff S.E., Marcantonio E.R. (2008). Chemokines Are Associated with Delirium after Cardiac Surgery. J. Gerontol. A Biol. Sci. Med. Sci..

[19] Kotfis K., Szylińska A., Listewnik M., Brykczyński M., Ely E.W., Rotter I. (2019). Diabetes and Elevated Preoperative Hba1c Level as Risk Factors for Postoperative Delirium after Cardiac Surgery: An Observational Cohort Study. Neuropsychiatr. Dis. Treat..

[20] Ferrer M., Bernadich O., Nava S., Torres A. (2002). Noninvasive Ventilation after Intubation and Mechanical Ventilation. Eur. Respir. J..

[21] Corlateanu A., Covantev S., Mathioudakis A.G., Botnaru V., Siafakas N. (2016). Prevalence and Burden of Comorbidities in Chronic Obstructive Pulmonary Disease. Respir. Investig..

[22] Nashef S.A.M., Roques F., Sharples L.D., Nilsson J., Smith C., Goldstone A.R., Lockowandt U. (2012). EuroSCORE II. Eur. J. Cardio-Thoracic Surg. Off. J. Eur. Assoc. Cardio-Thoracic Surg..

[23] Vogelmeier C.F., Criner G.J., Martinez F.J., Anzueto A., Barnes P.J., Bourbeau J., Celli B.R., Chen R., Decramer M., Fabbri L.M. (2017). Global Strategy for the Diagnosis, Management, and Prevention of Chronic Obstructive Lung Disease 2017 Report. GOLD Executive Summary. Am. J. Respir. Crit. Care Med..

[24] Huang X., Redfors B., Chen S., Liu Y., Ben-Yehuda O., Puskas J.D., Kandzari D.E., Merkely B., Horkay F., van Boven A.J. (2018). Impact of Chronic Obstructive Pulmonary Disease on Prognosis after Percutaneous Coronary Intervention and Bypass Surgery for Left Main Coronary Artery Disease: An Analysis from the EXCEL Trial. Eur. J. Cardio-Thoracic Surg..

[25] Szylińska A., Kotfis K., Listewnik M., Brykczyński M., Marra A., Rotter I. (2020). The Burden of Chronic Obstructive Pulmonary Disease in Open Heart Surgery—A Retrospective Cohort Analysis of Postoperative Complications: STROBE Compliant. Medicine.

[26] Lin W.-C., Chen C.-W., Lu C.-L., Lai W.-W., Huang M.-H., Tsai L.-M., Li C.-Y., Lai C.-H. (2019). The Association between Recent Hospitalized COPD Exacerbations and Adverse Outcomes after Percutaneous Coronary Intervention: A Nationwide Cohort Study. Int. J. Chron. Obstruct. Pulmon. Dis..

[27] Ried M., Unger P., Puehler T., Haneya A., Schmid C., Diez C. (2010). Mild-to-Moderate COPD as a Risk Factor for Increased 30-Day Mortality in Cardiac Surgery. Thorac. Cardiovasc. Surg..

[28] O’Boyle F., Mediratta N., Chalmers J., Al-Rawi O., Mohan K., Shaw M., Poullis M. (2013). Long-Term Survival of Patients with Pulmonary Disease Undergoing Coronary Artery Bypass Surgery. Eur. J. Cardio-Thoracic Surg..

[29] Leavitt B.J., Ross C.S., Spence B., Surgenor S.D., Olmstead E.M., Clough R.A., Charlesworth D.C., Kramer R.S., O’Connor G.T. (2006). Long-Term Survival of Patients with Chronic Obstructive Pulmonary Disease Undergoing Coronary Artery Bypass Surgery. Circulation.

[30] McAllister D.A., Wild S.H., MacLay J.D., Robson A., Newby D.E., MacNee W., Innes J.A., Zamvar V., Mills N.L. (2013). Forced Expiratory Volume in One Second Predicts Length of Stay and In-Hospital Mortality in Patients Undergoing Cardiac Surgery: A Retrospective Cohort Study. PLoS ONE.

[31] Saleh H.Z., Mohan K., Shaw M., Al-Rawi O., Elsayed H., Walshaw M., Chalmers J.A.C., Fabri B.M. (2012). Impact of Chronic Obstructive Pulmonary Disease Severity on Surgical Outcomes in Patients Undergoing Non-Emergent Coronary Artery Bypass Grafting. Eur. J. Cardio-Thoracic Surg..

[32] Adabag A.S., Wassif H.S., Rice K., Mithani S., Johnson D., Bonawitz-Conlin J., Ward H.B., McFalls E.O., Kuskowski M.A., Kelly R.F. (2010). Preoperative Pulmonary Function and Mortality after Cardiac Surgery. Am. Heart J..

[33] Evans A.S., Weiner M.M., Arora R.C., Chung I., Deshpande R., Varghese R., Augoustides J., Ramakrishna H. (2016). Current Approach to Diagnosis and Treatment of Delirium after Cardiac Surgery. Ann. Card. Anaesth..

[34] Cui X.-P., Jing Z.-Z., Song J.-F., Zhang P. (2019). A retrospective study on risk factors associated with postoperative delirium in elderly patients with spinal operation. Zhongguo Gu Shang.

[35] Tilouche N., Hassen M.F., Ali H.B.S., Jaoued O., Gharbi R., El Atrous S.S. (2018). Delirium in the Intensive Care Unit: Incidence, Risk Factors, and Impact on Outcome. Indian J. Crit. Care Med. Peer-Rev. Off. Publ. Indian Soc. Crit. Care Med..

[36] Austin V., Crack P.J., Bozinovski S., Miller A.A., Vlahos R. (2016). COPD and Stroke: Are Systemic Inflammation and Oxidative Stress the Missing Links?. Clin. Sci..

[37] Lahousse L., Tiemeier H., Ikram M.A., Brusselle G.G. (2015). Chronic Obstructive Pulmonary Disease and Cerebrovascular Disease: A Comprehensive Review. Respir. Med..

[38] Yousefshahi F., Samadi E., Paknejad O., Hagh E.B., Aminzadeh S. (2019). Effect of Hypoxemia in the Determination of Short-Term Prognosis of Coronary Artery Bypass Graft Patients: A Prospective Study. Anesthesiol. Pain Med..

[39] Kotfis K., Biernawska J., Zegan-Barańska M., Żukowski M. (2015). Peripheral Blood Lymphocyte Subsets (CD4+, CD8+ T Cells, NK Cells) in Patients with Cardiovascular and Neurological Complications after Carotid Endarterectomy. Int. J. Mol. Sci..

[40] Gosselt A.N., Slooter A.J., Boere P.R., Zaal I.J. (2015). Risk Factors for Delirium after On-Pump Cardiac Surgery: A Systematic Review. Crit. Care.

[41] Saller T., Petzold A., Zetterberg H., Kuhle J., Chappell D., von Dossow V., Klawitter F., Schurholz T., Hagl C., Reuter D.A. (2019). A Case Series on the Value of Tau and Neurofilament Protein Levels to Predict and Detect Delirium in Cardiac Surgery Patients. Biomed. Pap. Med. Fac. Univ. Palacky Olomouc Czechoslov..

[42] Alifier M., Olsson B., Andreasson U., Cullen N.C., Czyżewska J., Jakubów P., Sieśkiewicz A., Stasiak-Barmuta A., Hirnle T., Kornhuber J. (2020). Cardiac Surgery Is Associated with Biomarker Evidence of Neuronal Damage. J. Alzheimers. Dis..

[43] Gottesman R.F., Grega M.A., Bailey M.M., Pham L.D., Zeger S.L., Baumgartner W.A., Selnes O.A., McKhann G.M. (2010). Delirium after Coronary Artery Bypass Graft Surgery and Late Mortality. Ann. Neurol..

[44] Barr J., Fraser G.L., Puntillo K., Ely E.W., Gélinas C., Dasta J.F., Davidson J.E., Devlin J.W., Kress J.P., Joffe A.M. (2013). Clinical Practice Guidelines for the Management of Pain, Agitation, and Delirium in Adult Patients in the Intensive Care Unit. Crit. Care Med..

[45] Kotfis K., Zegan-Barańska M., Strzelbicka M., Safranow K., Żukowski M., Ely E.W. (2018). Validation of the Polish Version of the Critical Care Pain Observation Tool (CPOT) to Assess Pain Intensity in Adult, Intubated Intensive Care Unit Patients: The POL-CPOT Study. Arch. Med. Sci..

[46] Kotfis K., Strzelbicka M., Zegan-Barańska M., Safranow K., Brykczyński M., Żukowski M., Ely E.W. (2018). Validation of the Behavioral Pain Scale to Assess Pain Intensity in Adult, Intubated Postcardiac Surgery Patients: A Cohort Observational Study—POL-BPS. Medicine.

